# Vector competence across *Aedes aegypti* populations shaped by host-preference adaptation and host-pathogen interactions

**DOI:** 10.1128/jvi.00921-26

**Published:** 2026-07-23

**Authors:** Jorge A. Castro-Rodríguez, José Carlos Marín-Montesino, Silvia Ivonne Mora-Herrera, Fernando Ruíz-Jiménez, Miguel A. García-Knight

**Affiliations:** 1 Departamento de Inmunología, Instituto de Investigaciones Biomédicas, Universidad Nacional Autónoma de México 7180, Mexico City, Mexico; 2 Wellcome Sanger Institute 47665, Hinxton, United Kingdom; Indiana University Bloomington, Bloomington, Indiana, USA

**Keywords:** vector competence, *Aedes aegypti*, arbovirus, dengue

## Abstract

*Aedes aegypti* is the principal vector for yellow fever, dengue, Zika, and chikungunya viruses. Although this mosquito continues to expand its global range, its intrinsic ability to acquire, support the replication of, and transmit specific viruses—known as vector competence (VC)—varies across populations. A mechanistic understanding of this variation is a key goal of vector biology and is central to devising interventions to contain epidemics. In this review, we examine how evolutionary history fundamentally shaped VC in the sylvatic and human-adapted subspecies of *Ae. aegypti*. We discuss how the physiological processes underlying arbovirus infection define transmission potential and synthesize our understanding of the global heterogeneity in VC for the principal arboviruses transmitted by *Ae. aegypti*. Finally, we evaluate emerging insights into the molecular mechanisms that may drive this variation, with emphasis on the mosquito virome and the antiviral RNA interference pathways, and how these, in turn, help shape the genetic architecture associated with the intrinsic capacity to transmit arboviruses.

## INTRODUCTION

Mosquitoes are vectors for numerous arthropod-borne viruses (arboviruses) that are a huge global health burden. Chief among the vectors that transmit arboviruses is *Aedes aegypti*, the yellow fever mosquito, which transmits not only yellow fever virus (YFV) but also dengue (DENV), Zika (ZIKV), and chikungunya (CHIKV) viruses. Together, these pathogens are the most prevalent arboviruses worldwide, driving recurrent epidemics, with DENV alone being responsible for ~100 million annual clinical cases and ~30,000 deaths (1, 2). In addition to these pathogens, *Ae. aegypti* can also function as a secondary vector for understudied arboviruses, including Mayaro, Bunyamwera, and Cache Valley viruses (3–6). The expanding global distribution of *Ae. aegypti*, coupled with its capacity to sustain infections with arboviruses from multiple viral taxa—e.g., *Flaviviridae*, *Togaviridae*, and *Bunyavirales*—poses a significant risk for viral emergence and disease transmission.

**Fig 1:**
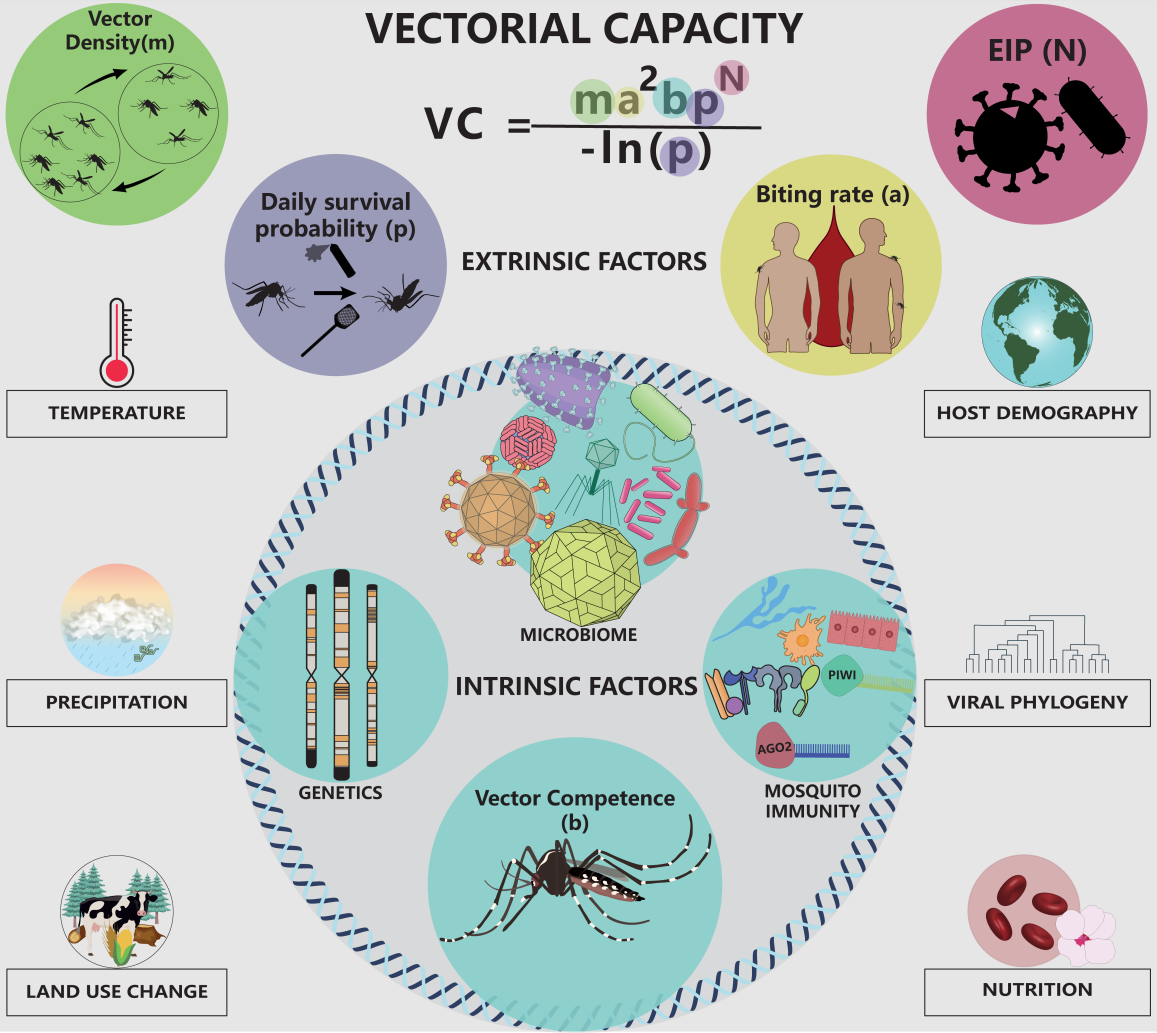
Factors that modulate vector competence in Ae. aegypti and its broader contribution to vectorial capacity. Vector competence (VC) is a complex phenotype underpinned by intrinsic factors: mosquito genetic background, the immune response, and the microbiome. In addition, extrinsic factors such as nutrition, land use change, climate, and pathogen-host interplay may influence the intrinsic determinants of VC. These extrinsic factors also influence vectorial capacity, a metric that quantifies the transmission potential of a vector population and secondary cases per unit time given the introduction of an infectious individual into a naïive population. It is defined in terms of five parameters, highlighted on the diagram: vector biting rate (a), vector density (m), probability of vector daily survival (p), vector competence (b), and pathogen extrinsic incubation period (EIP) (N).

The remarkable ability of *Ae. aegypti* to act as an efficient vector for human pathogens is fundamentally determined by its intrinsic capacity to acquire, support the replication of, and transmit specific viruses. This trait, termed vector competence (VC), is defined by host genetics and shaped by antiviral immunity and microbiome interactions (Fig. 1). Arbovirus transmission is further modulated by extrinsic, abiotic factors—including temperature and precipitation—and variables such as vector-host densities, mosquito longevity, and biting rate that define vectorial capacity (7, 8) (Fig. 1).

Evolutionary processes have further shaped vectorial capacity by influencing *Ae. aegypti* behavior. Specifically, ancestral sylvatic populations underwent a process of domestication in which females adapted to breeding in human dwellings and acquired anthropophilic biting preferences (9, 10). Notably, these adaptations were accompanied by an increase in VC (11–13). The invasive urban form of *Ae. aegypti* that emerged is now present in tropical and subtropical regions of over 100 countries in the Americas, Africa, Asia, Europe, and Oceania (14).

The determinants of vectorial capacity can vary across mosquito populations and over time. To project trajectories of *Ae. aegypti* invasiveness and arbovirus transmission risk, it is crucial to consider both the extrinsic factors that influence vectorial capacity—shaped, for instance, by anthropogenic environmental change (15, 16)—and the intrinsic factors that define variation in VC across vector populations. The mechanisms of VC for distinct arthropod vectors have been extensively reviewed (6, 17–22). With a focus on *Ae. aegypti*, this review examines the evolutionary and physiological foundations of VC variation, including the factors that influence vector competence after the ingestion of virus in a blood meal. In addition, we discuss recent advances in our understanding of the molecular mechanisms that modulate this variation and emphasize how these factors may intersect to influence the genetic basis of VC.

## *AE. AEGYPTI* POPULATION DIVERSITY AND EVOLUTIONARY ORIGINS

*Ae. aegypti* exists as two subspecies or ecotypes, the sylvatic *Ae. aegypti formosus* (*Aaf*) and the human specialist, globally invasive *Ae. aegypti aegypti* (*Aaa*) (23, 24). *Aaf* is a generalist subspecies and is present in a wide variety of habitats, including forested environments as well as towns and cities in Sub-Saharan Africa. Accordingly, females can take blood meals from non-human as well as human vertebrates and can lay eggs in natural containers like tree holes as well as human-manufactured containers. By contrast, *Aaa* is associated almost exclusively with human habitats where females feed on human blood meals and lay eggs in artificial water containers. Geographically, *Aaf* is restricted to Africa, while *Aaa* is found within as well as outside of Africa where it displays enhanced VC relative to *Aaf* for multiple arboviruses (11, 13, 25). Morphological differences accompany these distinctions: *Aaf* are predominantly darker than *Aaa* and lack pale scaling on the first abdominal tergite that is found on *Aaa* (26, 27). Variation in these traits, particularly in coastal Africa, has led to the suggestion that *Ae. aegypti* is polymorphic, and the subspecies classification is an oversimplification (23, 26). However, the existence of distinct subspecies is supported by population genetics (28–31), which has also helped explain some of the complex phenotypic variation in coastal Africa.

Continental Africa was long considered the most likely region where *Ae. aegypti* emerged (9, 23). However, phylogenetic analysis of the *Stegomyia* that populate the islands of the Southwest Indian Ocean instead supports the proximal divergence of ancestral *Ae. aegypti* in Madagascar (6, 32–34). Introduction to continental Africa is estimated to have occurred <85,000 years ago, followed by the westward and northward spread of the species after the end of the last glacial maximum (32, 35). An initial deep split in *Aaf* occurred between populations geographically restricted by distance and the East African Rift Valley. A more recent split in West African *Aaf* populations later led to the emergence of *Aaa* (28, 30).

Advances in sequencing technology and efforts to sample global genetic diversity (28–30) have enabled the use of whole genomes to disentangle the complex ancestry of *Ae. aegypti*. The *Aaf*/*Aaa* split has been dated to 2,300 (28) or 5,000 (30) years ago, when the West African Sahel region underwent aridification. These conditions would have provided a strong selective force on egg-laying behavior that influenced human specialization (10). Consistent reports suggest that global *Aaa* populations are monophyletic (10, 28, 30, 36). Crucially, specific coastal populations of *Ae. aegypti* in West Africa (e.g., Ngoye and Dakar in Senegal and Luanda in Angola) and in East Africa (e.g., domestic mosquitoes from Rabai, Kenya) exhibit shared *Aaa* and *Aaf* ancestry (10, 28, 37). Multiple studies have traced the *Aaa* ancestry component of domesticated Rabai mosquitoes to Asian populations (28, 29, 32, 36), indicating ongoing genetic introgression of invasive *Aaa* back to coastal East Africa. By contrast, West African populations possess *Aaa* ancestry that is basal to the invasive *Aaa* populations found worldwide (28, 30). These have been termed proto-*Aaa* and harbor the closest known ancestry of the globally invasive *Aaa* that diverged from *Aaf* in West Africa.

Historical records and phylogenetic estimates (23, 28, 31, 38) indicate that the transatlantic slave trade from West Africa served to spread proto-*Aaa* to the Americas, where the first reports of yellow fever epidemics date to the 16th century in the Caribbean (23, 39). Transport of humans on ships across the Atlantic further selected for invasive traits in *Aaa*, and rapid expansion into Asia, the Pacific, and Europe ensued (28). Mosquito control campaigns during the mid-20th century in the Americas eradicated founding *Aaa* populations from much of the continent, with exceptions in Argentina, Florida, and the West Indies (28, 31, 40, 41). *Aaa* was subsequently reintroduced into the Americas, possibly from Asian populations, following the cessation of mosquito eradication campaigns in the 1970s (28, 40). DENV transmission was later re-established, although the establishment of hyperendemic circulation of DENV serotypes in the Americas was delayed compared to Asia(42, 43).

How was enhanced VC in *Aaa* compared to *Aaf* selected, and how is it related to human specialization? The prevalence of mosquitoes infected with DENV (44), and other arboviruses (45), is low (<1%), even during an epidemic. It is therefore thought unlikely for arboviruses to represent a strong selective force influencing infection susceptibility (6, 46). Rather, enhanced VC in *Aaa* compared to *Aaf* appears to be part of a series of covarying traits associated with human specialization (10, 28). This began with adaptations in egg-laying behavior in response to the aridification of the Sahel, and traits like attraction to human odor and resistance to desiccationlater fine-tuned specialization (10). Recent studies have identified genome-wide adaptive signatures in global *Aaa* populations (29) and discrete genomic loci linked to human adaptation across *Aaf* populations (10). Together with pioneering early studies (11, 47), these findings provide important insights into the genetic basis of VC (discussed below). Given the genetic background of *Aaa* that emerged from this process, evolutionary forces acting on DENV (48–50), ZIKV (51, 52), and likely other viruses further influenced the infection process in the competent vector.

## VC AND THE PHYSIOLOGY OF ARBOVIRUS INFECTION

**Fig 2:**
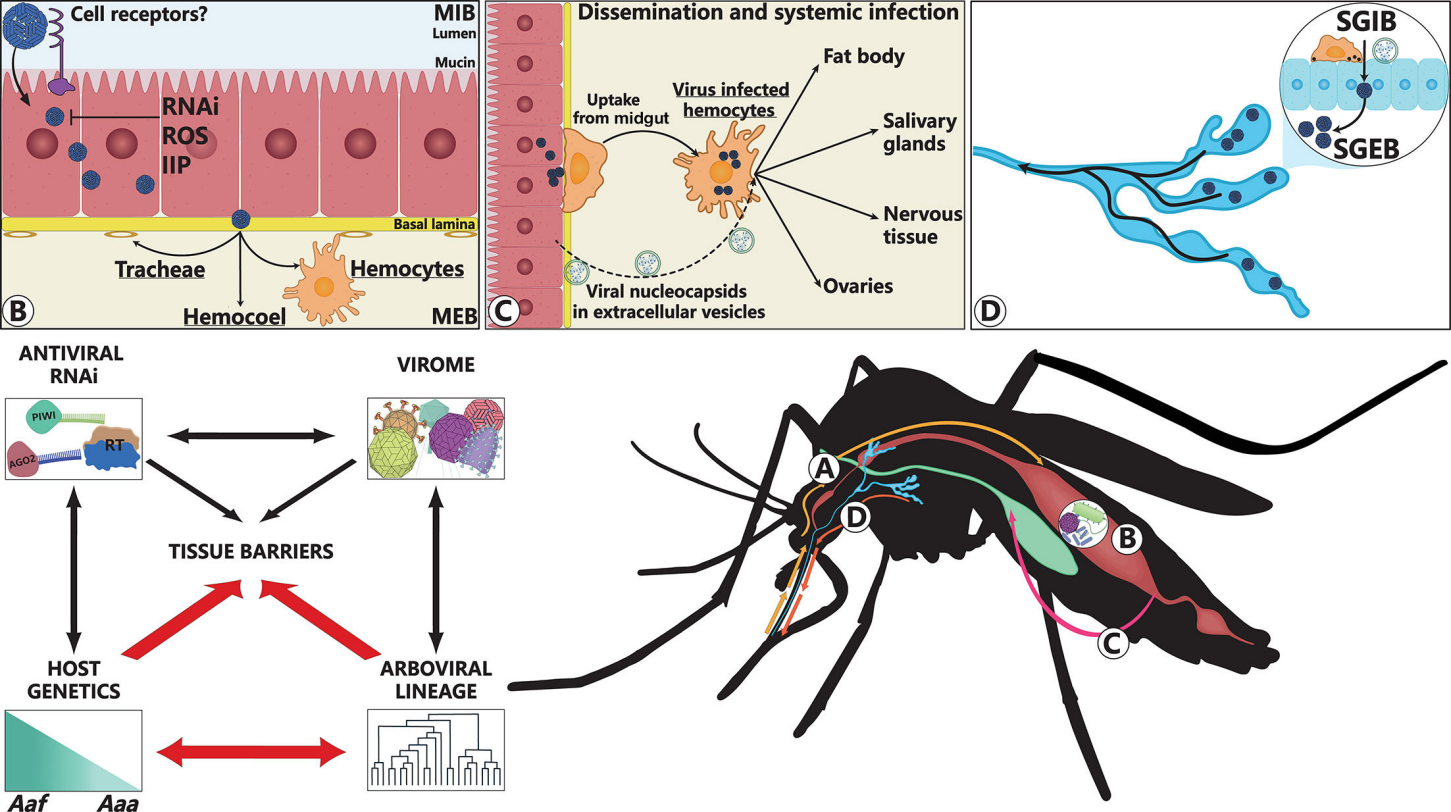
Arbovirus replication in *Ae. aegypti* and the interaction between intrinsic factors that modulate VC. The ability of *Ae. aegypti* to get infected and transmit arboviruses hinges on overcoming four tissue barriers: the midgut infection barrier (MIB), midgut escape barrier (MEB), salivary gland infection barrier (SGIB), and salivary gland escape barrier (SGEB). (**A**) Acquisition of viral particles following a blood meal is accompanied by bloodmeal-derived components that can promote cell permissiveness in the vertebrate host (not detailed graphically). (**B**) To overcome the MIB, the virus infects midgut epithelial cells through poorly defined receptors and attachment factors and interacts with the gut microbiome. Viruses are confronted by effectors of the mosquito’s innate immune pathways (IIP) such as the RNAi, Toll, IMD, JAK-STAT, and MAPK pathways, as well as by reactive oxygen species (ROS) (**C**) Following replication in midgut cells, the virus must cross the basal lamina to overcome the MEB and disseminate. Mature virions are inactivated by the acidity of the hemocoel. To overcome this, the virus can infect hemocytes or disseminate through extracellular vesicles carrying replication-competent viral nucleocapsids. Both processes enable viral spread to secondary tissues such as the fat body, ovaries, neural tissue, and salivary glands. (**D**) Infection of acinar cells at the SGIB enables further replication and the release of the virus into the salivary duct after overcoming the SGEB. Tissue barrier efficiency is influenced in large part by mosquito genetic background and the arboviral lineage through mosquito strain-by-virus genotype interactions. Tissue barriers are further influenced by the RNAi-mediated antiviral immune response and the mosquito virome, which may independently influence host genetics. Arbovirus genotype may additionally influence virome composition and vice versa.

A mechanistic understanding of arbovirus infection is central to defining VC. Following ingestion of a viraemic bloodmeal, the virus establishes systemic infection and is transmitted to a vertebrate host during a subsequent feed. This interval defines the extrinsic incubation period (EIP) (Fig. 2) and is constrained by anatomical barriers commonly referred to as the midgut infection and escape barriers (MIB and MEB) and the salivary gland infection and escape barriers (SGIB and SGEB) (53, 54). As such, these barriers describe physical constraints to viral dissemination whose molecular underpinnings are not fully understood. However, progression through these checkpoints depends on viral titer-dependent factors (e.g., antiviral immunity and microbiota) and independent factors (e.g., receptor incompatibility) (22, 53), as well as viral determinants (55).

The midgut epithelium is the first barrier to infection. In *Ae. aegypti*, it comprises ~5,000–10,000 cells, mainly enterocytes, along with enteroendocrine and intestinal stem cells, and enteroblasts (56–58). Single-cell analyses revealed functional heterogeneity in these subsets and bloodmeal-induced shifts in cell composition (56, 58). Although ZIKV RNA has been detected in enterocytes and enteroendocrine cells (59), the specific subsets initially permissive to entry and amplification with distinct arboviruses remain unclear.

Arboviruses typically enter midgut cells via receptor-mediated endocytosis, and infection has been shown to be dependent on flavivirus envelope (E) proteins (60). Several candidate receptors for DENV in *Ae. aegypti* have been proposed, including a 67 kDa protein (possibly enolase) (61–63), prohibitin (64), and EPrRec (65), although their roles as true receptors versus attachment factors (66) remain debated (53, 57). Viral factors such as flavivirus NS1 may also enhance infection by promoting attachment (57) and suppressing mosquito antiviral responses, including reactive oxygen species (67).

After replication in the midgut, viruses must overcome the MEB by traversing the basal lamina (BL) (68), a proteinaceous extracellular matrix that excludes virions (53). Repeated blood meals are linked to BL disruption, enhanced viral egress, and a shortened EIP (69). Viral escape across the BL may also involve tracheal cells (70) or cardiac tissue (71). The MEB constitutes a major bottleneck that shapes viral diversity (72). Following dissemination, viral replication occurs in secondary tissues—including the fat body, neural tissue, ovaries, and salivary glands—thereby amplifying the viral population (73). These tissues are separated from the midgut by the hemocoel. A recent study demonstrated that flavivirus virions are inactivated by the acidic environment of the hemocoel and that viral dissemination is achieved through extracellular vesicles (EVs) that package replication-competent viral nucleocapsids (60). Notably, interactions between the mosquito valosin-containing protein and the viral capsidmediated nucleocapsid incorporation into EVs and dictated specificity for flavivirus infection across vector species. Accordingly, mosquito phagocytic hemocytes amplify arboviruses through direct infection—thereby preventing virion exposure to the hemocel—and facilitate viral dissemination via interactions with midgut cells and secondary tissues (73) (Fig. 2). Despite these insights, the relative contribution of these dissemination pathways across distinct arbovirus infections remains to be fully understood.

Finally, disseminated virus must infect acinar cells of the salivary glands. Even after systemic dissemination, differences in attachment factor expression or local immunity can prevent transmission (74, 75). A SGIB occurs when glands remain uninfected despite systemic spread; a SGEB occurs when virus fails to enter saliva. If virus reaches saliva, salivary components can enhance mammalian infection (76)—for example, *Ae. aegypti* salivary serine proteases promote DENV infection by modifying the extracellular matrix, increasing viral attachment (77).

## VC VARIATION ACROSS *AE. AEGYPTI* POPULATIONS

The transmission dynamics of *Ae. aegypti*-vectored arboviruses vary globally. To evaluate the contribution of VC to this variation, experimental infection assays measure viral dissemination across tissue barriers using different mosquito populations (reared as laboratory colonies for varying generations) and arbovirus isolates. Here, we summarize these findings alongside epidemiological trends for the major *Ae. aegypti*-transmitted arboviruses.

### YFV

YFV belongs to the *Flaviviridae* family and was the first virus identified to be transmitted by *Ae. aegypti* (78). It circulates in Africa and South America, is absent from Asia and the Pacific, and, despite the existence of an effective vaccine, caused >100,000 symptomatic cases and up to 51,000 deaths in 2018 alone (79). YFV originated in Africa and diverged into two genotypes, the ancestral East African genotype and the West African genotype (38, 80), with the latter giving rise to two South American genotypes (38, 80). YFV is maintained in sylvatic transmission cycles, and outbreaks of sylvatic strains are reported across Africa and recently in Brazil (80, 81) and Colombia (82, 83). By contrast, urban epidemics are mostly associated with West Africa, such as those that occured in Angola and the DRC in 2015–2016 (84), and were documented in the Americas before the mosquito eradication campaigns of the mid-20th century (39). Urban transmission is associated with *Ae. aegypti,* whereas sylvan *Aedes* spp. (e.g., *Ae. africanus*) and *Haemagogus* spp. and *Sabethes* spp. mediate the sylvatic cycle with non-human primates (NHP) in Africa and South America, respectively. Assessments of the VC in *Ae. aegypti* for YFV have therefore been key to evaluating urban YFV transmission risk across regions.

Early studies described a viral titer-dependent reduction in the susceptibility of sylvan *Aaf* strains from Uganda (25) and Senegal (11) compared to domestic African strains and non-African strains, possibly because of a MGEB (85). More recently, Dickson et al. (86) demonstrated heterogeneity in midgut infection in mosquitoes from across Senegal and frequent refractoriness to disseminated infection, although this varied depending on the YFV isolate. Agha et al. (87) used an East African YFV genotype to compare infection susceptibility and dissemination in mosquitoes from three urban locations in Kenya. with distinct *Aaf* and *Aaa* ancestry. Strikingly, although midgut infection was observed in 22% of orally challenged individuals, dissemination was not detected in any of the 805 individuals tested. Conversely, *Ae. aegypti* colonies from Central Africa (88, 89) and Cape Verde (90) sustained infections with a Senegalese YFV strain with dissemination in up to 50% of individuals and viral detection in saliva, indicating a reduced MGEB.

In the Americas, studies have compared VC in *Ae. aegypti* strains from across the continent. In Brazil, mosquitoes from Santos and Minas Gerais infected with a Brazilian YFV isolate could sustain disseminated and salivary gland infections (35% and 25% of individuals tested, respectively) (91). In addition, Couto-Lima et al. (88) compared mosquitoes from the endemic and YFV-free regions of Brazil and found that strains from non-endemic regions could sustain and transmit isolates of American and West African YFV at similar or increased levels than those from endemic regions. In the Caribbean, mosquitoes collected in Guadeloupe (92) and across the region (41) were susceptible to African and American YFV strains, with disseminated infection and viral detection in saliva.

Together, these studies suggest the following: (i) VC variation for YFV between *Aaa* and *Aaf* (although complex ancestries in coastal Africa are rarely considered), (ii) a possible midgut escape barrier contributing to VC variation, although explicit barrier characterization remains limited; (iii) a strain-by-virus-genotype effect on susceptibility, as seen with other arboviruses; (iv) contrasting susceptibilities in East and West African mosquitoes that influence urban outbreaks, and (v) competent vector populations across South America and the Caribbean that pose a risk for urban YFV emergence. Considering that competent vectors are present in Asia and North America, it remains unclear which factors limit contemporary YFV outbreaks in these areas.

### DENV

DENV is a flavivirus comprising four antigenically distinct serotypes (DENV1–4), each divided into multiple genotypes (93). The evolutionary origin of DENV remains unclear, although independent zoonotic spillovers from NHP reservoirs in Asia have been suggested as sources (94). Both sylvatic and urban cycles are involved in DENV transmission, with the latter mediated primarily by *Ae. aegypti* and, to a lesser extent, by *Ae. albopictus* (95). The sylvatic cycle has been documented in Asia (involving all four serotypes) and in West Africa (involving DENV-2), and transmission is associated with diverse *Aedes* spp. mosquitoes (94).

DENV epidemiology is well characterized in many regions of the Americas and Asia, where year-round and seasonal hyperendemic transmission is sustained in multiple countries. By contrast, reports of outbreaks and severe disease have historically been less frequent in Africa, although endemic DENV transmission is thought to occur in 34 countries and all four serotypes have been isolated on the continent (96). Moreover, repeated large-scale outbreaks have been documented in East (97–100) and West Africa (101–103), involving high infection burdens and multiple serotype co-circulation. Conversely, in inland East Africa, such as Uganda, DENV transmission appears limited, with low seroprevalence across age groups between 2016 and 2019 (104, 105).

The apparent epidemiological distinctions in Africa have been associated with underdeveloped surveillance systems in some settings, mis- or underdiagnosis, host genetic ancestry, and cross-reactive flavivirus immunity (106). Low VC in sylvatic *Ae. aegypti* populations have also been assessed as a potential contributing factor. Several studies report reduced DENV infection in sylvatic *Aaf* compared to invasive *Aaa* (107–110). For example, viral dissemination was observed in 75% of individuals with *Aaa* ancestry compared to 32% in *Aaf* strains from Senegal, with an MGEB implicated in the reduced susceptibility (12); similar observations were reported in Kenya (111). Additionally, infrequent saliva infections in *Aaf* mosquitoes with disseminated infections have suggested a role for SGIB or SGEB (90). In contrast, assessments of seven mosquito strains from across Africa with varying degrees of *Aaa* ancestry found considerable heterogeneity in DENV susceptibility (112). Although reduced DENV susceptibility was generally observed in *Aaf*, strain-by-virus genotype interaction was apparent across six DENV isolates, and some vector strains showed infection rates exceeding those of *Aaa* controls. As with African populations, VC variation was observed in *Aaa* populations from Brazil at different geographic scales (113, 114), albeit in a background of high susceptibility where strain-by-virus genotype variation has also been documented (115, 116). Overall, these findings challenge the strict dichotomy of DENV susceptibility in *Aaf* and *Aaa* and suggest that viral genotype, the microbiome, and the antiviral response interact with the genetic background in *Aaf* and *Aaa* to modulate VC for DENV.

### ZIKV

ZIKV is closely related to DENV and exists as a single serotype with two main lineages: the Asian and African lineages (117). The evolutionary origin of ZIKV has been traced to Uganda, where it was first isolated from a sentinel NHP and an *Ae. africanus* mosquito in the Zika forest (117). Like DENV, ZIKV circulates in both sylvatic and urban transmission cycles, and urban transmission is mainly mediated by *Ae. aegypti*. Although sylvatic transmission was thought to only occur in Africa (118), recent studies support the emergence of a sylvatic transmission cycle in neotropical primates and sylvatic mosquito species in South America (119–121).

Large epidemics caused by the Asian lineage have occurred in Asia, the Pacific and Indian Ocean islands, and notably in the Americas, where the virus was introduced in 2015 and became endemic. In Africa, however, reports are generally limited to sporadic epizootic cases, with the exception of recent Asian lineage outbreaks in Cape Verde (122) and Angola (123). Like DENV, reduced VC in *Aaf* has been assessed as a contributing factor to these dynamics. Analysis of *Ae. aegypti* colonies from Africa, Asia, and the Americas infected with eight ZIKV strains representing global diversity consistently showed reduced susceptibility of African colonies to infection and reduced viral transmission to susceptible mice (13). Furthermore, the proportion of *Aaa* ancestry in mosquito populations (124), including those in Cape Verde (125), correlated with infection susceptibility. As with DENV, variation in ZIKV susceptibility was observed in the *Aaa* genetic background (126, 127), with high VC apparent in populations from Guadeloupe and French Guiana (13, 128). VC was also associated with viral strain (129–131), with enhanced infection observed with African vs Asian lineage isolates in mosquito strains from the Americas (124, 132) and West Africa (133), suggesting possible strain-by-virus genotype interactions as described for DENV. Importantly, structural genes in the African lineage ZIKV enhanced viral transmission in *Ae. aegypti* by facilitating viral internalization, particularly influencing the SGIB and SGEB (55).

### CHIKV

CHIKV is an arthritogenic alphavirus that causes an estimated 35.3 million infections a year globally (134). It is of African origin, initially identified in Tanzania in 1953 (135). Three major lineages exist: the East-Central-South-African (ECSA), West African, and Asian lineages. While the West African lineage is mainly enzootic, involves sylvan *Aedes* spp. vectors, and leads to cyclical, geographically limited outbreaks, the Asian and ECSA lineages have expanded their range since 2004, causing urban outbreaks across Africa, Asia, Oceania, and the Americas (136). Urban transmission is associated mainly with *Ae. aegypti* and *Ae. albopictus*, with the latter able to extend transmission to temperate and rural regions.

Many VC studies for CHIKV have focused on comparisons between *Ae. aegypti* and *Ae. albopictus*. Notably, following an ECSA lineage outbreak in La Réunion Island in 2006, VC was increased in *Ae. albopictus* due to an adaptive A226V substitution in the E1 viral glycoprotein (137) and epistatic mutations (138, 139), although these mutations did not significantly alter VC in *Ae. aegypti*. In contrast, the Asian lineage is genetically constrained in its ability to adapt to *Ae. albopictus,* and VC is comparatively higher for this lineage (as well as for the ECSA lineage lacking E1A226V) in *Ae. aegypti* (138). A broad comparison of VC in mosquitoes from across the Americas found high infection and dissemination levels (>90%) in both species following oral exposure to Asian and ECSA lineage viruses (140). Notably, saliva viremia was detected in high proportions in specific populations of both species (>80%), although this varied substantially across sites, suggestive of variation in SGIB/SGEB. Overall, the global expansion and high invasiveness of *Ae. albopictus*, combined with viral adaptation, represent a significant risk for sustained and expanded CHIKV transmission worldwide.

Few studies have compared VC for CHIKV in *Aaf* and *Aaa* populations. Agha et al. (141) compared VC in mosquitoes from the coastal city of Mombasa, Kenya, to strains from the interior of the country. Oral infection was dose-dependent, suggestive of an MGIB, and ~60% of individuals from all locations sustained infections at high bloodmeal viral titers. Similarly, VC in mosquitoes was compared between urban and peri-urban sites in Ouagadougou, Burkina Faso (133), a locality of complex *Aaf*/*Aaa* ancestry in which the urban population had increased white scaling of the 1st abdominal tergite (10). Overall, infection rates were similar to strains from Kenya (65%), although the dissemination rate was reduced in peri-urban vs urban populations (85% vs 45%), together with the viral load in the body. In addition, *Aaf* populations with complex ancestry from Cape Verde were highly competent with high infection rates (100%) and virus detection in saliva in up to 70% of individuals with experimental oral infections (90). Overall, these studies suggest that global *Ae. aegypti* populations exhibit high vector competence, although transmission efficiency varies due to largely unknown factors. Comparative studies using strains with defined ancestry remain limited.

## MOLECULAR MECHANISMS THAT MODULATE VC

As detailed in Fig. 2, intrinsic modulators of VC include vector genetics, antiviral immunity, and microbiome interactions. The impact of microbial interactions—including bacterial endosymbionts and fungal microorganisms—as well as the broader antiviral immune response on VC has been reviewed extensively (142–144). Here, we focus on the mosquito virome and antiviral RNAi and how they may impact the genetic basis for VC.

### Getting to the core of the *Ae. aegypti* virome

Arboviruses infect arthropods (including insects) and vertebrates, but most viruses that make up the mosquito virome are insect-specific viruses (ISVs) that do not replicate in vertebrate cells and are predominantly transmitted vertically (145). Metagenomic analyses have shown that mosquitoes are generally infected with several ISVs that can be from diverse families or orders of mainly RNA viruses (146–148). These include taxa in which arboviruses are also found, such as *Bunyavirales, Flaviviridae, Rhabdoviridae,* and *Togaviridae,* and novel taxa. Notably, single or combinations of certain ISVs can modulate arbovirus replication and influence VC (149–151). For instance, DENV and ZIKV replication is restricted in *Ae. aegypti* infected with cell fusing agent virus (CFAV) (152) and Nhumirim virus (153). These studies suggest that ISVs could potentially be employed in the management of arbovirus transmission (154, 155). However, key aspects of ISV biology, including factors shaping mosquito virome composition and the mechanisms by which ISVs influence arbovirus replication, remain poorly understood.

With respect to the latter, current findings highlight both patterns and inconsistencies in virome structure. Analyses of distinct species from discrete localities identified ISVs in high abundance in the same species (156–160), suggesting that this taxonomic level plays a major role in defining virome composition. Indeed, virome stability across developmental stages (159, 161) and mosquito populations led to the suggestion of the existence of a core virome (159). However, the utility of this term is under debate (154, 162, 163), and analyses of *Culex* spp. (164) found little consistency in virome composition across individuals. Nevertheless, in *Ae. aegypti,* abundant ISVs include CFAV (*Flaviviridae*), Phasi-Charoen-like virus (PCLV; *Phenuviridae*), Humaita Tubiacanga virus (HTV; unclassified), and Aedes anphevirus (AAV; *Ximnoviridae*) (149, 165–167). Additionally, at least 27 ISVs have been identified in *Ae. aegypti* globally (146). Comparisons of lab strains and field caught *Aedes* spp. mosquitoes show increased diversity in the latter, suggesting that in addition to host species, environmental factors (149, 161, 168) and possibly ISV-arbovirus interactions (166) also influence virome diversity.

ISVs modulate VC by either antagonizing or promoting arbovirus replication. The mechanisms of antagonism are not well understood, although superinfection exclusion, through competition for resources within a co-infected cell or by the induction of a cross-reactive immune response, may play a role (169). Recently, combining global virome diversity analysis and a murine infection model, Olmo et al. reported a positive effect of PCLV/HTV coinfection on DENV and ZIKV transmission by *Ae. aegypti* (149). The authors identified histone H4 as a proviral factor and suggest that PCLV/HTV prevents its downregulation during arbovirus infection, leading to enhanced DENV and ZIKV replication. The effect of PCLV and HTV could not be decoupled, however, and the authors suggest that HTV could independently mediate the effect. Accordingly, PCLV replication was found to increase after a mosquito bloodmeal in midgut and fat body cells (56), and in the latter, this increase was associated with upregulation of a newly proposed DENV restriction factor, cytochrome P450 (170). These apparent contrasting effects of PCLV warrant further investigation.

These compelling findings suggest that the prevalence of PCLV, HTV, and other ISVs may influence VC across *Ae. aegypti* populations. Indeed, in individual mosquitoes from Southeast Brazil, those with DENV infections were enriched with PCLV/HTV coinfections compared to uninfected individuals (149). Interestingly, recent assessments of global virome diversity in *Ae. aegypti* found that invasive *Aaa* mosquitoes from the Americas and Asia harbor similar ISVs, with PCLV and HTV showing the highest prevalence (149, 167). However, differences across latitudes were observed in the Americas, with PCLV and HTV largely absent from mosquitoes from the United States, and AAV absent from mosquitoes from Brazil (167). By contrast, *Aaf* populations showed the lowest level of ISV diversity, with no PCLV/HTV coinfections and the presence of distinct ISVs such as Formosus virus (*Rhabdoviridae*) (149, 167). It remains unclear whether this variation reflects environmental modulation of virome composition, differences in genetic ancestry, or both, and if these differences contribute to the VC phenotypes in *Aaa* and *Aaf*.

### An adaptive immune response with heritable features?

Unlike in vertebrates, arboviruses cause persistent infections in mosquitoes. Viral replication has been reported to negatively impact mosquito survival in some virus-vector combinations, although the limited data on *Aedes* mosquitoes infected with flaviviruses suggests little or no detectable harm to the organism (171). This occurs in the face of an antiviral immune response, which limits viral replication but does not clear the infection. In insects, one of the main antiviral defense pathways, which is central to this tolerance phenotype (172, 173), is the RNA interference (RNAi) pathway, a conserved, sequence-specific gene-silencing mechanism triggered by double-stranded RNA (dsRNA). It regulates gene expression and transposons and, in antiviral defense, targets viral RNA genomes to control infection (174).

RNAi effectors are ~20–30 nucleotide (nt) small-interfering RNAs (siRNAs) that guide the silencing machinery to complementary nucleic acids. Conserved Argonaut (Ago) family proteins drive this process. Three main small RNA classes exist—microRNAs, siRNAs, and P-element-induced wimpy testis (PIWI)-interacting RNAs (piRNAs)—each with distinct roles. In mosquitoes and flies, 21-nt siRNAs derived from viral dsRNA intermediates are central to antiviral defense and require Dicer-2 and Ago-2 (175–177). The piRNA pathway, known canonically for transposon silencing in germline cells, also contributes to antiviral defense in mosquitoes through the activity of 25–30-nt piRNAs (178–180). *Ae. aegypti* has an expanded set of PIWI-clade proteins (eight vs three in *D. melanogaster*), that appear to have undergone specialization for the antiviral response in somatic tissues. Deep sequencing after DENV (178), CHIKV (181), and Sindbis virus (SINV) (182) infections identified both virus-derived siRNAs and piRNAs in mosquito cells, although the biogenesis pathway for the latter remains unclear.

Both flies and mosquitoes have an amplification pathway for virus-specific small RNAs (172, 183–185). This involves the formation of viral DNA (vDNA) from the genomes of non-retroviral RNA viruses. vDNA serves as a stable template for producing secondary virus-derived small RNAs. This process is essential for viral tolerance: blocking vDNA formation causes uncontrolled viral replication and mosquito death (172). vDNA synthesis depends on reverse transcriptase activity from endogenous retrotransposons, as shown by inhibition of vDNA formation with azidothymidine. Retrotransposons generate circular DNA intermediates, and vDNA-retrotransposon chimeras have been detected in *Ae. aegypti (172, 186*). Although the exact mechanism remains unclear, vDNA likely forms through recombination between viral RNA and retrotransposon RNA during reverse transcription, possibly involving the Dicer-2 helicase domain (187). These findings suggest that *Ae. aegypti* uses a conserved pathway to develop specific, stable antiviral protection with the hallmarks of adaptive immunity in vertebrates. In *D. melanogaster*, hemocytes play a central role in this response (183). They take up viral dsRNA via scavenger receptors (188), promote vDNA formation, and produce viral siRNAs that are distributed systemically in exosome-like vesicles. This mechanism enables antiviral protection in distant, uninfected tissues, providing systemic immunity (183). Whether *Ae. aegypti* hemocytes specialize in vDNA formation, and secondary viral siRNA production is not clear.

DNA sequences from non-retroviral RNA viruses can exist not only as episomal vDNA but also as endogenous viral elements (EVEs). These are horizontally acquired fragments of viral sequences integrated into host genomes. In *Ae. aegypti,* hundreds of non-retroviral (nr) EVEs, derived principally from ISVs, are found enriched at genomic loci associated within piRNA clusters (189–192). In addition, EVE-derived piRNAs (generally found in an antisense orientation with regard to their targets) are observed in small RNA sequencing libraries of *Aedes* spp. (149, 167, 173). Whether these piRNAs mediate functional antiviral responses has been a key question.

In the Aag2 cell line, which is persistently infected with PCLV and CFAV, genomic piRNA clusters were found with cognate EVEs that produce antisense piRNAs specific for these ISVs (173, 186). Knockdown of core components of the piRNA pathways increased PCLV and CFAV replication. Moreover, insertion of EVE targets in a sense direction in a recombinant SINV reporter reduced viral replication up to 100-fold compared to an antisense EVE target (173). Subsequent reports *in vivo* further suggest that piRNAs derived from a CFAV EVE limit CFAV replication in *Ae. aegypti* ovaries, possibly influencing vertical transmission (193). In the case of alphaviruses, experimental infections found specific antiviral responses to be inherited over several generations in a vDNA-dependent manner (194, 195). These findings suggest that vertical vDNA transmission or genome integration events could lead to transgenerational antiviral immunity, although how this operates across arboviruses is not well understood.

### Genetic determinants of VC and genomic plasticity

VC is a complex phenotype likely influenced by multiple genetic loci (21). Mapping of the genetic basis for VC variation has, therefore, been investigated through quantitative trait loci (QTL) analysis. Mosquito strains with contrasting MIB and MEB to DENV-2 were initially used in genetic crosses to map QTLs associated with these barriers (47, 196, 197). Across studies, nine QTLs were identified at discrete regions in all three chromosomes. In addition, five genomic regions containing QTLs associated with VC for parasitic pathogens were identified that co-clustered with those for DENV-2 (198), implying that these may be important for non-specific immunity or common infection pathways. However, low resolution, due to a limited number of genomic markers, precluded the identification of specific VC-associated genetic components in early studies.

The ability to sequence whole mosquito genomes has transformed the genetic analysis of VC, including QTL analyses (199). Determinants of VC for ZIKV were recently studied using ∼230,000 single-nucleotide polymorphisms (SNPs) identified on a genome-wide scale (13). ZIKV-susceptible and -resistant *Aaa* and *Aaf* strains, respectively, were crossed, and regions encoding alleles underlying ZIKV susceptibility were mapped to nine QTLs found between megabase 37.0 and 344.8 on chromosome 2. Despite low resolution, this region coincides with a locus associated with DENV dissemination (199) and contains 49/185 protein-coding genes identified as being part of the adaptive signature in *Aaa* that underpins domestication (29). These signatures were identified by comparing 511 *Aaf* and 123 *Aaa* whole genomes representing global diversity. Gene ontology analysis showed that the signatures are related to chemosensory, metabolic, regulatory, neuronal, and immunological functions. Notably, >65% had polymorphisms in *Aaf* populations, indicating that most adaptive alleles originated from the standing genetic variation found in Africa. Parallel efforts to assess adaptation to humans across *Aaf* populations in Africa further identified discrete regions harboring 16,782 SNPs associated with human specialization, which clustered over all three chromosomes (10). These studies highlight the potential for continued adaptation in *Aaf* under changing climatic conditions that may impact VC. They also provide a key resource to help narrow down the location of genetic determinants that underpin VC.

The generation of chromosome-level long-read genomes coupled with Hi-C approaches to detect chromatin interactions has further advanced assessments of the genetic basis for VC (199–201). This has enabled the study of chromosomal inversions in *Ae. aegypti* and their role in adaptation (201). Chromosomal inversions impact malaria infectivity in *Anophelinae* malaria vectors (202), but their role in *Ae. aegypti* evolution had been relatively unexplored. Characterization of inversion polymorphisms in global *Aaf* and *Aaa* populations found that *Aaf* genomes, particularly from West Africa, harbor an increased number of inversions (200, 201), with some possibly impacting QTLs previously associated with broad VC for pathogens (198).

Beyond revealing chromosomal inversions, long-read assemblies enable repetitive genome stretches to be resolved and the characterization of EVEs in their genomic context. The 1.25 Gb genome of *Ae. aegypti* is highly repetitive, with ~65% composed of transposable elements (TEs), including 11.7% LTR retrotransposons (199, 203). Estimates of nrEVEs in reference assemblies vary from 129 (190) to 472 (186), mostly derived from *Flaviviridae*, *Rhabdoviridae,* and *Chuviridae*. These are predominantly located within transcriptionally active piRNA clusters interspersed with TEs that likely shuffle and fragment viral sequences (204). A prominent *flamenco*-like piRNA cluster on chromosome 2 plays a major role in antisense EVE-derived piRNA production (190). While most nrEVEs originate from ISVs, populations of field mosquitoes harbor distinct EVEs absent from reference genomes (191, 192), including from pathogens like DENV-1 and Liao ning virus (*Reoviridae*) (29). Despite these insights, the frequency of EVE integration in somatic vs germline tissues remains unclear, and whether nrEVEs contribute to antiviral defense and VC or primarily reflect TE-mediated genomic byproducts is under debate (190, 203, 204).

## CONCLUSIONS

Arboviruses are climate-sensitive pathogens, and warming temperatures are projected to increase transmission risk in Africa, Europe, and North America (16, 205). Accordingly, unprecedented *Ae. aegypti*-vectored arbovirus outbreaks across endemic regions have been recorded in recent years (206, 207). The development of novel interventions to complement advances in vector management (208) will require a mechanistic understanding of the intrinsic factors that shape VC.

Genomic analyses of global mosquito populations have shown how evolutionary history and adaptive processes shaped VC in *Aaf* and *Aaa* (10, 28–30). These data sets provide a key resource to experimentally determine the contribution of QTLs—particularly prevalent on chromosome 2—on susceptibility phenotypes and discern common modulators across arboviruses and those specific to viral species and lineages. Standardized protocols and reagents to assess VC in experimental settings are needed to facilitate this process (6). The identification of an *Aaa* molecular signature in 68 genes encompassing 483 non-synonymous variants (29) will be instrumental in further deciphering the complex genetic histories of populations on the African coasts and crucially monitoring *Aaa* introductions across the continent. Understanding the ecological factors that favor *Aaa* recolonization in Africa is a key priority.

We are only beginning to appreciate the extent to which the *Ae. aegypti* virome and EVEome vary globally, and how this impacts VC in *Aaa* and *Aaf* populations. For instance, whether variation in the “core” virome along the North/South axis in the Americas impacts arbovirus transmission remains unknown. Ongoing characterization of ISVs has also reshaped viral taxonomy and may provide new avenues for the development of genetic tools to modulate VC (154, 167). In addition, the impact of ISVs and arbovirus infection on genome plasticity through EVE acquisition and modulation of the RNAi response is also an area of intense interest. Key questions remain regarding the frequency of viral endogenization and whether the process is compartmentalized in specific tissues or cell types such as hemocytes. Considering the low prevalence of arboviruses in mosquito populations, it also remains unclear whether selection acts on specific EVEs that may impact the formidable ability of *Ae. aegypti* to vector arboviruses.
